# Erythrocyte sedimentation rate and albumin as markers of inflammation are associated with measures of sarcopenia: a cross-sectional study

**DOI:** 10.1186/s12877-019-1253-5

**Published:** 2019-08-27

**Authors:** Vera A. van Atteveld, Jeanine M. Van Ancum, Esmee M. Reijnierse, Marijke C. Trappenburg, Carel G. M. Meskers, Andrea B. Maier

**Affiliations:** 1 Department of Human Movement Sciences, @AgeAmsterdam, Vrije Universiteit Amsterdam, Amsterdam Movement Sciences, Amsterdam, The Netherlands; 2 Department of Medicine and Aged Care, @AgeMelbourne, The Royal Melbourne Hospital, The University of Melbourne, Centre for Medical Research building, Melbourne, 300 Grattan Street, Parkville, Victoria 3010 Australia; 3 Department of Internal Medicine, VU University Medical Center, Amsterdam, The Netherlands; 4 Department of Internal Medicine, Amstelland Hospital, Amstelveen, The Netherlands; 5 Department of Rehabilitation Medicine, VU University Medical Center, Amsterdam Movement Sciences, Amsterdam, The Netherlands

**Keywords:** Blood sedimentation, Albumin, White blood cells, Inflammation, Muscular atrophy, Outpatients, Sarcopenia

## Abstract

**Background:**

Chronic inflammation is considered to affect physical performance, muscle strength and muscle mass, i.e. measures of sarcopenia. We need to identify a marker of inflammation that is univocally associated with measures of sarcopenia. We aimed to associate three markers of inflammation, erythrocyte sedimentation rate, albumin and white blood cell count, with measures of sarcopenia in geriatric outpatients.

**Methods:**

Data from the Centre Of Geriatrics Amsterdam cohort was used. Geriatric outpatients at the VU university medical centre in Amsterdam were recruited based on referral between January 1st 2014 and the 31st of December 2015. Erythrocyte sedimentation rate, albumin and white blood cell count were assessed from venous blood samples. Measures of sarcopenia included physical performance by measuring gait speed with the 4 meter walk test, duration of the timed up and go test and of the chair stand test, muscle strength by assessing handgrip strength using handheld dynamometry and skeletal muscle mass by performing bioelectrical impedance analysis. Multivariable linear regression analyses were performed to assess the associations between erythrocyte sedimentation rate, albumin, white blood cell count and measures of sarcopenia.

**Results:**

A total of 442 patients (mean age 80.8 years, SD 6.7, 58.1% female) were included. A higher erythrocyte sedimentation rate was significantly associated with lower gait speed (β = − 0.005; 95% CI = − 0.007, − 0.003), longer duration of timed up and go test (Ln β = 0.006; 95% CI = 0.003, 0.010), longer duration of chair stand test (Ln β = 0.005; 95% CI = 0.002, 0.008), lower handgrip strength (β = − 0.126; 95% CI = − 0.189, − 0.063) and lower relative skeletal muscle mass (β = − 0.179; 95% CI = − 0.274, − 0.084). Lower albumin levels were significantly associated with lower gait speed (β = − 0.020; 95% CI = − 0.011, − 0.028) and handgrip strength (β = − 0.596; 95% CI = − 0.311, − 0.881). Associations remained significant after adjustment for age, sex and number of morbidities. No significant associations were found for white blood cell count and measures of sarcopenia.

**Conclusions:**

In geriatric outpatients**,** erythrocyte sedimentation rate was associated with all three measures of sarcopenia, underpinning the potential role of inflammation in sarcopenia.

**Electronic supplementary material:**

The online version of this article (10.1186/s12877-019-1253-5) contains supplementary material, which is available to authorized users.

## Background

Sarcopenia, low physical performance, muscle strength and muscle mass, recently redefined by the European Working Group on Sarcopenia in Older People (EWGSOP2) [1], is a highly prevalent disease ranging up to 15% in older persons of 60 to 70 years old, and between 40 to 50% in individuals of 80 years and older [2, 3]. Sarcopenia is associated with impaired functional status [4], which is related to adverse health outcomes such as nursing home admission and mortality [5]. Many factors have been identified that are involved in the pathophysiology of sarcopenia, such as malnutrition, hormonal changes (lower levels of growth hormone and sex hormones), physical inactivity and chronic inflammation [6].

Inflammation is thought to be an important risk factor for sarcopenia, as it induces a catabolic state in the muscles [7]. Inflammatory markers that have been investigated in relation to sarcopenia showed contradicting results, including C-reactive protein (CRP), erythrocyte sedimentation rate (ESR), albumin, white blood cell (WBC) count, cytokines (e.g. Tumor Necrosis Factor-α (TNF-α), Interleukine-6 (IL-6)) and its soluble receptors [8–16]. TNF-α, IL-6, CRP, albumin, WBC count and ESR were found to be associated with low physical performance, muscle strength or muscle mass in older people [8, 9, 13, 14, 16], but other studies failed to find this association [10, 11, 13, 17]. To date, TNF-α, IL-6, albumin and CRP have shown a relation with the development of low muscle strength or muscle mass over time [8, 13–15], however, the current body of evidence fails to reveal a marker of chronic inflammation that is univocally associated with measures of sarcopenia in older people [18, 19].

The aim of the present paper is to associate ESR, albumin and WBC count as markers of inflammation, with measures of sarcopenia i.e. physical performance, muscle strength and muscle mass in a cohort of geriatric outpatients. The number of studies evaluating geriatric outpatients is limited.

## Methods

### Study design

The Centre Of Geriatrics Amsterdam (COGA) cohort encompasses 572 patients who were referred to the geriatric outpatient clinic of the VU University Medical Center in Amsterdam, the Netherlands, between January 1st 2014 and the 31st of December 2015. Study inclusion was based on referral by general practitioners due to either mobility, cognitive problems or functional decline. A comprehensive geriatric assessment was performed by a geriatric nurse, including measurements of physical and cognitive performance. The assessment was followed by a consultation of a geriatrician. Blood samples were collected in 505 patients, of which ESR, albumin or WBC count and at least one physical test were available in 442 patients. No exclusion criteria were applied. The Medical Ethical Committee of the VU University Medical Center approved the study (reference number 2017.582). As this cross-sectional study was based on regular care, the need for individual informed consent was waived.

### Patient characteristics

Relevant characteristics such as age, sex, living status, education, alcohol use, smoking, medication use, medical history, cognitive status, weight, height and functional status were obtained in the abovementioned comprehensive geriatric assessment. Use of anti-inflammatory medication was defined as the use of prednisone, prednisolone, methotrexate, mesalazine, hydroxychloroquine, infliximab or non-steroid anti-inflammatory drugs (NSAID). Medical history that was counted as morbidities included angina, dementia, depression, diabetes type 1 or 2, heart failure, hypercholesterolemia, hypertension, hyperthyroidism, hypothyroidism, malignancy, myocardial infarction, renal insufficiency, osteoporosis, Parkinson’s disease, transient ischemic attack (TIA), cerebral infarction, arrhythmias, joint prosthesis, joint disease or obstructive pulmonary disease. Cognitive status was tested using the mini-mental state examination (MMSE) [20]. Functional status included independency in activities of daily living (ADL) measured by the Katz index [21] with a higher score indicating more dependency, use of a walking aid, immobility for more than 1 week in the preceding 3 months, maximal walking distance and experiencing a fall in the preceding year. Hemoglobin level in mmol/L was measured using non-hemolyzed EDTA-anticoagulated whole blood analyzed using the sodium lauryl sulphate (SLS) detection method. SLS binds with heme, causing a color change that is measured photometrically (Sysmex XN9000, Sysmex BV, Etten-Leur, the Netherlands).

### Inflammatory markers

ESR in millimeter/hour (mm/hr) was measured using non-hemolyzed EDTA-anticoagulated whole blood that was analyzed following the Westergren method (StaRRsed Auto Compact VERA109900, Mechatronics BV, Hoorn, the Netherlands). The Westergren method is de reference method of the International Council for Standardization in Hematology (ICSH) [22].

Albumin in g/L was measured in serum using a colorimetric test with bromcresol purple. Bromcresol purple binds selectively with albumin and the color change is measured photometrically (Cobas 8000 modular analyzer series, Roche Netherlands BV, Woerden, the Netherlands).

WBC count in E^9^/L was measured using non-hemolyzed EDTA-anticoagulated whole blood with fluorescence flow cytometry (Sysmex XN9000, Sysmex BV, Etten-Leur, the Netherlands).

### Measures of sarcopenia

Gait speed was assessed by the 4 meter walk test at usual pace [23], and expressed in meters/second (m/s). The timed up and go (TUG) test as a measure of functional mobility [24], and the chair stand test (CST) as an indicator of lower limb strength [25] were both expressed in seconds (s).

Handgrip strength (HGS) was measured by a hand dynamometer in a standing position with a stretched arm (angle of 180 degrees) (Jamar hand dynamometer, Sammons Preston, Inc., Bolingbrook, IL) and expressed in kilograms (kg). Maximal handgrip strength out of six attempts (three left and three right) was used [26].

Muscle mass was measured in a standing position using direct segmental multi-frequency bioelectrical impedance analysis (DSM-BIA; InBody 720; Biospace Co.,Ltd., Seoul, Korea), which is a validated tool to assess body composition [27]. Patients with a pacemaker or defibrillator were excluded from this measurement. Two parameters were extracted from the DSM-BIA measurements: relative skeletal muscle mass (RMM) in percent calculated as [skeletal muscle mass in kg/weight*100] and appendicular lean mass (ALM) divided by height^2^ in kg/m^2^ calculated as [ALM/height^2^]. DSM-BIA measurements were available in 68 patients as these measurements were added to the comprehensive geriatric assessment in a later stage of the inclusion period.

### Statistical analyses

For normally distributed variables, mean values and standard deviations (SD) were presented, for skewed variables medians and interquartile ranges (IQR), and for categorical variables the number and percentage. The associations between the independent variables (ESR, albumin and WBC count) and the dependent variables (measures of sarcopenia) were analyzed using multivariable linear regression. Results were presented as β with 95% confidence intervals (95% CI) and *p* values; significance level was set at α = 0,05. Skewed variables (TUG test and CST), were log-transformed using the natural logarithm. The log-transformed β can be interpreted as follows: [(e^β^ − 1). 100] equals the percentage of change in the dependent variable with every unit increase of the independent variable. Age, sex and number of morbidities were identified as possible confounders. An additional sensitivity analysis was performed, excluding patients who used anti-inflammatory medication (*n* = 36). A second additional analysis was performed, comparing ESR, albumin levels and WBC count for patients with low or normal measures of sarcopenia based on the EWGSOP2 definition cut-off points, using logistic regression analyses adjusted for age, sex and number of comorbidities. For the statistical analysis, IBM SPSS statistics 25 for Windows was used. For visual presentation, ESR tertiles were calculated and bar charts were made using GraphPad Prism version 7.00 for Windows, GraphPad Software, La Jolla California USA.

## Results

Patient characteristics are shown in Table 1. The mean age of the population was 80.8 years (SD: 6.7), 58.1% was female, 87.2% of the patients were living independently and the median number of morbidities was 3 (IQR: 2–4).

**Table 1:** Patient characteristics

Characteristics	*n*	Total (*n* = 442)
General		
Age, years	442	80.8 (6.7)
Sex, female, n (%)	442	257 (58.1)
Living independently, n (%)	421	367 (87.2)
Education, years, median [IQR]	380	10 [9–13]
Use of alcohol, n (%)	408	255 (62.5)
Current smoking, n (%)	410	43 (10.5)
Polypharmacy, n (%)*	441	257 (58.3)
Anti-inflammatory medication, n (%)†	440	36 (8.2)
Number of morbidities, median [IQR]	441	3 [2–4]
MMSE score, median [IQR]	425	26 [22–28]
Weight, kg	413	70.2 (13.5)
Height, cm	414	166.6 (9.7)
BMI, kg/m^2^	408	25.3 (4.2)
Hemoglobin, mmol/L	435	8.6 (0.9)
Anemia, n (%)	435	83 (19.1)
Functional status		
Katz ADL score, median [IQR]	399	0 [0–1]
Use of walking aid, n (%)	408	169 (41.4)
Immobile > 1 week in last 3 months, n (%)	387	59 (15.2)
Maximal walking distance, > 1 km, n (%)	391	140 (35.8)
Fall in past year, n (%)	404	246 (60.9)
Inflammatory markers		
ESR, mm/hr., median [IQR]	408	11 [6–22]
Albumin, g/L, median [IQR]	433	37.8 [35.7–39.7]
WBC count, E^9^/L, median [IQR]	442	7.2 [6.0–8.7]
Measures of sarcopenia		
Gait speed, m/s	419	0.8 (0.3)
TUG test, s, median [IQR]	303	14.9 [11.5–19.0]
CST, s, median [IQR]	356	13.6 [11.4–18.6]
HGS, kg	423	22.2 (9.6)
RMM, %	68	36.7 (5.4)
ALM/height^2^, kg/m^2^	68	7.1 (1.2)

All variables are presented as mean (standard deviation), unless otherwise specified

*ADL* Activities of daily living, *ALM* Appendicular lean mass, *BMI* Body mass index, *CST* Chair stand test, *ESR* Erythrocyte sedimentation rate, *HGS* Handgrip strength, *IQR* Interquartile range, *MMSE* Mini-mental state examination, *RMM* Relative skeletal muscle mass, *TUG* Timed up and go, *VAS* Visual analogue scale, *WBC* White blood cell

*Polypharmacy: use of > 4 medication

†Anti-inflammatory medication: use of prednisone, prednisolone, methotrexate, mesalazine, hydroxychloroquine, infliximab or non-steroid anti-inflammatory drugs

**Fig. 1:**
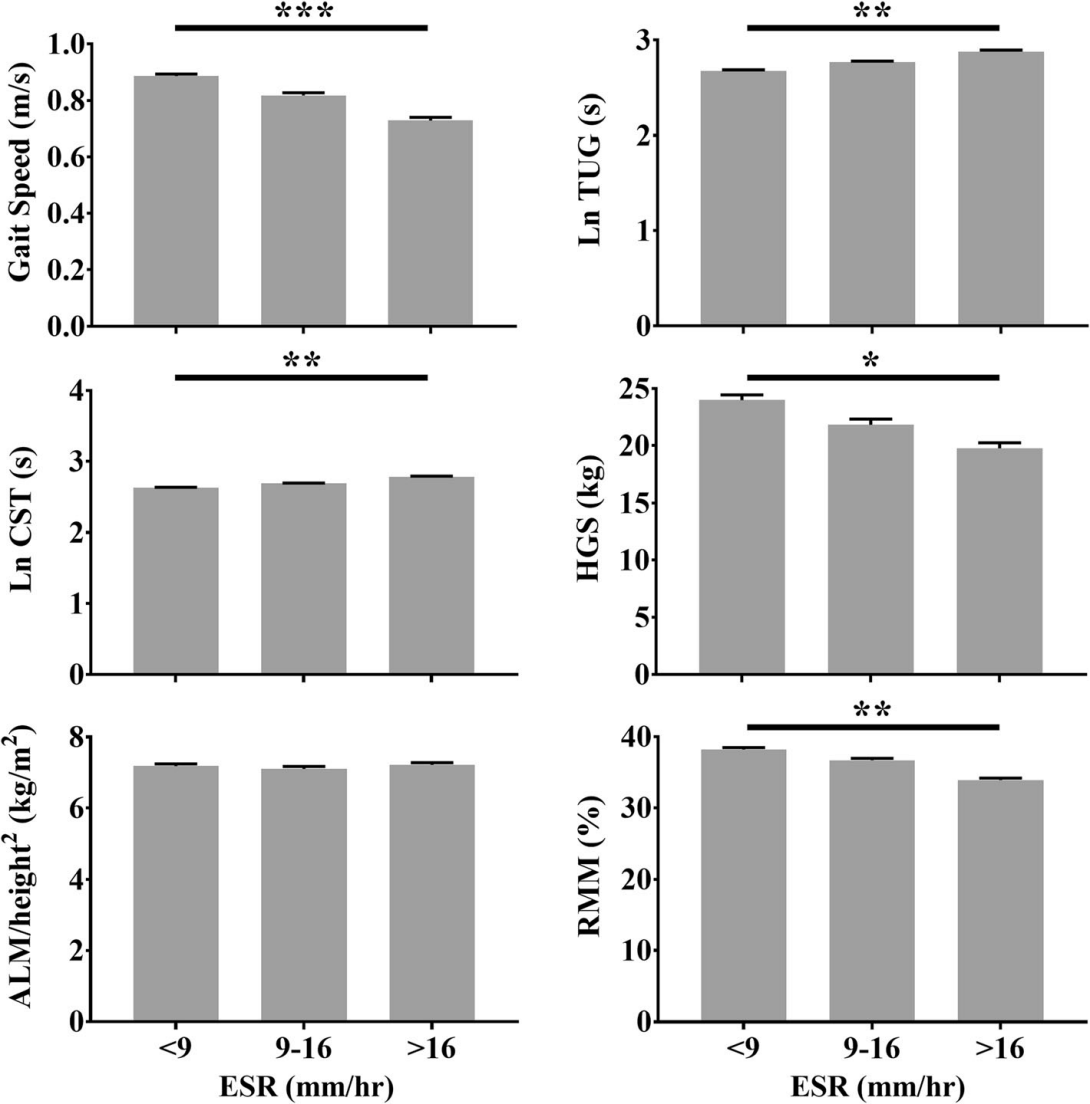
Erythrocyte sedimentation rate tertiles and measures of sarcopenia. Bars: Estimated means, adjusted for age, sex and number of morbidities. Error bars: 1 standard error. Level of significance based on linear regression: * *p* ≤ 0.05; ** *p* < 0.01; *** *p* < 0.001. *ALM* Appendicular lean mass, *CST* Chair stand test, *ESR* Erythrocyte sedimentation rate, *HGS* Handgrip strength, *Ln* Natural logarithm, *RMM* Relative skeletal muscle mass, *TUG* Timed up and go

The associations between ESR, albumin and WBC count with physical performance are shown in Table 2. Higher ESR was significantly associated with lower gait speed and longer duration of the TUG test and CST. After adjustment for age, sex and number of morbidities, these associations remained significant. Higher ESR was significantly associated with lower HGS and lower RMM both in the crude model as well as in the adjusted model. No association was found between ESR and ALM/height^2^. Lower albumin was significantly associated with lower gait speed and handgrip strength in the crude as well as in the adjusted model. No associations were found between albumin and duration of the TUG test and CST, RMM and ALM/height^2^. Higher WBC count was significantly associated with lower gait speed and handgrip strength, however, after adjustment the associations were no longer significant. Figure 1 shows the tertiles of ESR with measures of sarcopenia.

**Table 2:** Outcomes of linear regression analysis of the association between ESR, albumin and WBC count with measures of sarcopenia

		Gait speed, m/s	Ln TUG test, s	Ln CST, s	HGS, kg	RMM, %	ALM/height^2^, kg/m^2^
ESR	Crude β 95% CI	−0.005 −0.007, −0.003	0.006 0.003, 0.010	0.005 0.002, 0.008	−0.126 −0.189, −0.063	−0.179 −0.274, −0.084	−0.000 −0.022, 0.022
	*p* value	**0.000**	**0.000**	**0.001**	**0.000**	**0.000**	1.000
	Adjusted β 95% CI	−0.004 −0.006, −0.002	0.005 0.002, 0.008	0.005 0.002, 0.008	−0.052 −0.102, −0.001	−0.115 −0.196, −0.034	0.010–0.011, 0.031
	*p* value	**0.000**	**0.002**	**0.003**	**0.046**	**0.006**	0.328
Albumin	Crude β 95% CI	0.020 0.011, 0.028	−0.020 −0.034, −0.006	−0.012 −0.025, 0.001	0.596 0.311, 0.881	0.233–0.244, 0.711	0.002–0.101, 0.106
	*p* value	**0.000**	**0.006**	0.062	**0.000**	0.333	0.962
	Adjusted β 95% CI	0.014 0.006, 0.022	−0.013 −0.027, 0.000	−0.009 −0.022, 0.004	0.455 0.225, 0.684	0.375–0.014, 0.764	− 0.007 −0.102, 0.088
	*p* value	**0.001**	0.057	0.166	**0.000**	0.059	0.886
WBC count	Crude β 95% CI	−0.011 −0.021, − 0.002	0.011–0.005, 0.026	0.002–0.012, 0.016	−0.354 −0.679, − 0.029	−0.654 −1.362, 0.055	− 0.105 −0.263, 0.053
	*p* value	**0.018**	0.178	0.785	**0.033**	0.070	0.188
	Adjusted β 95% CI	−0.005 −0.014, 0.004	0.006–0.009, 0.021	− 0.001 −0.015, 0.013	−0.112 −0.371, 0.146	− 0.430 −1.017, 0.156	−0.071 −0.214, 0.071
	*p* value	0.299	0.409	0.921	0.393	0.147	0.322

*ALM* Appendicular lean mass, *β* Beta, *CI* Confidence interval, *CST* Chair stand test, *ESR* Erythrocyte sedimentation rate, *HGS* Handgrip strength, *Ln* Natural logarithm, *RMM* Relative skeletal muscle mass, *TUG* Timed up and go, *WBC* White blood cell. Adjusted model: adjusted for age, sex, number of morbidities. Bold indicates a statistical significant outcome

Results did not change significantly when excluding patients using anti-inflammatory medication (Additional file 1: Table S1). Additional file 2: Table S2 shows the median [IQR] levels of ESR, albumin and WBC count for patients with low or normal measures of sarcopenia. ESR and albumin were different between low or normal gait speed, TUG, CST and HGS. For low or normal ALM/height^2^, no significant difference was found. WBC count was not different for any of the measures of sarcopenia.

## Discussion

In a cohort of geriatric outpatients higher ESR was associated with worse physical performance measured as lower gait speed, longer duration of the TUG test and CST, lower muscle strength expressed as HGS, and lower muscle mass expressed as RMM. Lower albumin was associated with lower HGS and gait speed. WBC count was not associated with any measure of sarcopenia.

A single and reliable marker for sarcopenia has not been found yet. This may be due to the many pathways that are involved in causing sarcopenia such as the endocrine pathway and the inflammation-mediated pathway that the present study examined. Inflammation can be measured using different markers, such as IL-6, TNF-α, CRP, butyryl-cholinesterase and oxidized low-density lipoprotein [28]. Results from previous studies are contradictory regarding the association of inflammatory markers and sarcopenia. ESR was found to be elevated in a sarcopenic group of 36 geriatric outpatients compared to a non-sarcopenic group of 36 geriatric outpatients [9], as well as in a group of 101 geriatric inpatients with sarcopenia compared to a non-sarcopenic group of 458 geriatric inpatients admitted to the rehabilitation ward [29]. However, in a cohort of 200 independent-living older adults, ESR was comparable between sarcopenic and healthy adults [17]. Lower albumin was associated with a decline in muscle mass after three to 5 years in community-dwelling men and women [14, 15]. Other inflammatory markers such as TNF-α, IL-6, WBC count, butyryl-cholinesterase and CRP were found to be correlated with poorer physical performance, muscle strength or muscle mass in geriatric outpatients and community-dwelling older adults [8, 13, 16, 30–32]. However, other studies failed to find an association between IL-6 and physical performance, nor confirm the findings for IL-6 and CRP and muscle strength and muscle mass in community-dwelling older people [10, 11, 13]. The current study included a broad range of measures of sarcopenia, and found that ESR is associated with three measures of physical performance (gait speed, duration of the TUG test and the CST), as well as with muscle strength and muscle mass. Albumin was found to be associated with two measures of sarcopenia: gait speed and handgrip strength.

The inverse associations between inflammatory markers and measures of sarcopenia may be explained by ageing. Ageing is associated with a state of chronic low-grade inflammation [33], the main source of pro-inflammatory molecules being adipose tissue [7]. Increased levels of inflammatory markers can lead to skeletal muscle proteolysis activation, raised insulin resistance lowering the inhibition of protein catabolism by insulin [34], and eventually atrophy of skeletal muscle [7]. This may explain why no significant associations were found for WBC count. Higher WBC count is most typically associated with acute infections rather than with chronic low grade inflammation [35], and is less commonly studied in this perspective as opposed to other makers [36].

In the current study, ESR was not significantly associated with ALM/height^2^. On the contrary, the association with RMM, a percentage of body mass, was significant in all statistical models. In previous studies, ALM/height^2^ and RMM are commonly used in diagnosis of sarcopenia, but there is no consensus on which measure defines low muscle mass best [3]. The significant association between ESR and RMM may reflect the tight interaction between fat mass and muscle mass in the sense that a higher fat mass leads to a chronic pro-inflammatory state which is subsequently associated with sarcopenia [37].

### Strengths and limitations

Strengths of the present study were the large sample of patients in a relevant study population of geriatric outpatients, and the use of different outcome measures encompassing measures of sarcopenia. There are a few limitations to this study to report. The study samples of outpatients with muscle mass measures were small, as BIA measurements were added to the protocol in a later stage. The small sample size might have reduced the power of the associations with muscle mass. ESR, albumin and WBC count are not specific markers of chronic inflammation. ESR, albumin levels and WBC count can also change as a result of other (non-chronic) conditions. Other markers of inflammation such as CRP and butyryl-cholinestarase were not available in our database, therewith the analyses were limited to ESR, albumin and WBC count [28, 32]. Furthermore, the cross-sectional study design cannot prove causality, so our findings need to be confirmed in a longitudinal study.

## Conclusions

In summary, in a cohort of geriatric outpatients, ESR, a marker of chronic inflammation, was associated with all three measures of sarcopenia: physical performance, muscle strength and muscle mass. Albumin was associated with handgrip strength and gait speed. No associations were found for WBC count. These results underpin a role of chronic inflammation in sarcopenia in geriatric outpatients, a population that has not been extensively studied to date.

## Additional files

Additional file 1: **Table S1.** Outcomes of linear regression analyses of the associations between ESR, albumin and WBC count with measures of sarcopenia, without patients who use anti-inflammatory medication. (DOCX 15 kb)

Additional file 2: **Table S2.** ESR, albumin and WBC count compared for cut-off points for measures of sarcopenia according to EWGSOP 2. (DOCX 16 kb)

## Data Availability

The datasets used and/or analyzed during the current study are available from the corresponding author on reasonable request.

## Abbreviations

ADL: Activities of daily living
ALM: Appendicular lean mass
CI: Confidence interval
COGA: Centre of geriatrics Amsterdam
CRP: C-reactive protein
CST: Chair stand test
DSM-BIA: Direct segmental multi-frequency bioelectrical impedance analysis
ESR: Erythrocyte sedimentation rate
HGS: Handgrip strength
ICSH: International council for standardization in hematology
IL-6: Interleukine-6
IQR: Interquartile range
MMSE: Mini-mental state examination
NSAID: Non-steroid anti-inflammatory drugs
RMM: Relative skeletal muscle mass
SD: Standard deviation
TIA: Transient ischemic attack
TNF- α: Tumor necrosis factor α
TUG: Timed up and go
WBC: White blood cell
