# Supplementary material for: Erythrocyte sedimentation rate and albumin as markers of inflammation are associated with measures of sarcopenia: a cross-sectional study
Source: BMC Geriatr. 2019 Aug 27;19:233. doi: 10.1186/s12877-019-1253-5 (PMC6712841; doi:10.1186/s12877-019-1253-5)
Supplement: Supplementary file 1 — Table S1. Outcomes of linear regression analyses of the associations between ESR, albumin and WBC count with measures of sarcopenia, without patients who use anti-inflammatory medication. (DOCX 15 kb) [file 12877_2019_1253_MOESM1_ESM.docx]

| **Supplementary Table 1.** Outcomes of linear regression analysis of the association between ESR, albumin and WBC count with measures of sarcopenia, without patients who use anti-inflammatory medication. | | | | | | | |
| --- | --- | --- | --- | --- | --- | --- | --- |
|  |  | Gait speed, m/s | Ln TUG test, s | Ln CST, s | HGS, kg | RMM, % | ALM/height^2^, kg/m^2^ |
| ESR | Crude β 95% CI | -0.005  -0.007, -0.003 | 0.006  0.002, 0.010 | 0.006  0.003, 0.009 | -0.128  -0.197, -0.059 | -0.181  -0.282, -0.079 | 0.001  -0.023, 0.025 |
|  | *p* value | **0.000** | **0.001** | **0.000** | **0.000** | **0.001** | 0.927 |
|  | Adjusted β 95% CI | -0.004  -0.006, -0.002 | 0.005  0.001, 0.008 | 0.005  0.002, 0.008 | -0.057  -0.112, -0.003 | -0.117  -0.205, -0.028 | 0.011  -0.012, 0.033 |
|  | *p* value | **0.000** | **0.006** | **0.001** | **0.040** | **0.011** | 0.356 |
|  |  |  |  |  |  |  |  |
| Albumin | Crude β 95% CI | 0.018  0.008, 0.027 | -0.014  -0.030, 0.003 | -0.011  -0.025, 0.003 | 0.596  0.280, 0.912 | 0.056  -0.493, 0.606 | -0.057  -0.174, 0.061 |
|  | *p* value | **0.000** | 0.103 | 0.138 | **0.000** | 0.839 | 0.337 |
|  | Adjusted β 95% CI | 0.013  0.003, 0.022 | -0.007  -0.022, 0.009 | -0.008  -0,022, 0,006 | 0.462  0.211, 0.712 | 0.235  -0.196, 0.666 | -0.070  -0.174, 0.034 |
|  | *p* value | **0.007** | 0.378 | 0,278 | **0.000** | 0.279 | 0.185 |
|  |  |  |  |  |  |  |  |
| WBC count | Crude β 95% CI | -0.009  -0.019, 0.001 | 0.009  -0.008, 0.025 | -0.002  -0.017, 0.012 | -0.347  -0.691, -0.003 | -0.327  -1.221, 0.567 | -0.096  -0.292, 0.101 |
|  | *p* value | 0.077 | 0.292 | 0.761 | **0.048** | 0.466 | 0.333 |
|  | Adjusted β 95% CI | -0.003  -0.013, 0.007 | 0.005  -0.011, 0.020 | -0.005  -0.020, 0.010 | -0.134  -0.405, 0.138 | 0.060  -0.655, 0.755 | -0.087  -0.262, 0.089 |
|  | *p* value | 0.553 | 0.565 | 0.504 | 0.334 | 0.867 | 0.325 |
| ALM: Appendicular lean mass. β: Beta. CI: Confidence interval. CST: Chair stand test. ESR: Erythrocyte sedimentation rate. HGS: Handgrip strength. Ln: Natural logarithm. RMM: Relative skeletal muscle mass. TUG: Timed up and go. WBC: White blood cell. Adjusted model: adjusted for age, sex, number of morbidities. Bold indicates a statistical significant outcome. Patients who use anti-inflammatory medication (n=36) are excluded. | | | | | | | |
