# Supplementary material for: Erythrocyte sedimentation rate and albumin as markers of inflammation are associated with measures of sarcopenia: a cross-sectional study
Source: BMC Geriatr. 2019 Aug 27;19:233. doi: 10.1186/s12877-019-1253-5 (PMC6712841; doi:10.1186/s12877-019-1253-5)
Supplement: Supplementary file 2 — Table S2. ESR, albumin and WBC count compared for cut-off points for measures of sarcopenia according to EWGSOP 2. (DOCX 16 kb) [file 12877_2019_1253_MOESM2_ESM.docx]

| **Supplementary Table 2.** ESR, albumin and WBC count compared for cut-off points for measures of sarcopenia according to EWGSOP 2. | | | | | | | |
| --- | --- | --- | --- | --- | --- | --- | --- |
|  |  | EWGSOP2 cut-off points  for measures of sarcopenia | | | |  |  |
|  |  | n | Low  Median [IQR] | n | Normal  Median [IQR] | OR (95%CI) | *p*-value |
| Gait speed | ESR, mm/hr | 198 | 14.5 [8.0-26.0] | 189 | 9.0 [5.0-17.0] | 1.03 (1.01-1.05) | **0.001** |
|  | Albumin, g/L | 206 | 37.2 [35.2-39.1] | 204 | 38.6 [36.6-40.4] | 0.91 (0.84-0.97) | **0.005** |
|  | WBC count, mmol/L | 206 | 7.5 [6.3-8.9] | 205 | 6.8 [5.8-8.3] | 1.00 (0.93-1.07) | 0.882 |
| TUG | ESR, mm/hr | 61 | 16.0 [10.0-26.5] | 216 | 10.0 [6.0-16.0] | 1.04 (1.01-1.06) | **0.002** |
|  | Albumin, g/L | 64 | 37.6 [35.5-39.9] | 233 | 38.1 [36.4-40.2] | 0.96 (0.88-1.06) | 0.444 |
|  | WBC count, mmol/L | 63 | 7.3 [6.1-8.5] | 233 | 6.8 [5.8-8.2] | 0.98 (0.89-1.09) | 0.727 |
| CST | ESR, mm/hr | 127 | 14.0 [6.0-26.0] | 203 | 10.0 [6.0-18.0] | 1.02 (1.00-1.04) | **0.017** |
|  | Albumin, g/L | 134 | 37.6 [35.4-39.3] | 215 | 38.2 [36.3-40.4] | 0.94 (0.87-1.01) | 0.075 |
|  | WBC count, mmol/L | 134 | 7.2 [6.0-8.7] | 216 | 6.8 [5.9-8.2] | 1.00 (0.93-1.08) | 0.995 |
| HGS | ESR, mm/hr | 146 | 13.0 [6.0-24.3] | 243 | 11.0 [6.0-21.0] | 1.01 (0.99-1.02) | 0.308 |
|  | Albumin, g/L | 158 | 37.3 [35.2-39.4] | 256 | 38.1 [36.5-40.0] | 0.91 (0.85-0.98) | **0.009** |
|  | WBC count, mmol/L | 157 | 7.4 [6.2-8.9] | 256 | 6.9 [5.9-8.6] | 1.05 (0.97-1.13) | 0.213 |
| ALM/  height^2^ | ESR, mm/hr | 14 | 8.5 [2.3-13.8] | 49 | 11.0 [6.0-21.0] | 0.95 (0.88-1.03) | 0.185 |
|  | Albumin, g/L | 16 | 37.4 [34.8-38.4] | 52 | 37.8 [36.0-39.7] | 1.01 (0.93-1.10) | 0.777 |
|  | WBC count, mmol/L | 16 | 7.6 [6.2-8.6] | 51 | 7.0 [6.0-8.1] | 1.04 (0.77-1.40) | 0.822 |
| ALM: Appendicular lean mass. CST: Chair stand test. ESR: Erythrocyte sedimentation rate. HGS: Handgrip strength. OR: Odds Ratio. TUG: Timed up and go. WBC: White blood cell. Odds ratio adjusted for age, sex, number of morbidities. Bold indicates a statistical significant outcome. | | | | | | | |
